# Reduction of airborne and surface-borne bacteria in a medical center burn intensive care unit using active, upper-room, germicidal ultraviolet (GUV) disinfection

**DOI:** 10.1017/ice.2023.223

**Published:** 2024-03

**Authors:** Linda D. Lee, Louise Lie, Michael Bauer, Berenice Bolanos, Russell N. Olmsted, Jay K. Varma, Jorge P. Parada

**Affiliations:** 1 UV Angel, Grand Haven, Michigan; 2 Loyola University Medical Center, Maywood, Illinois; 3 Trinity Health, Livonia, Michigan; 4 Weill Cornell Medical College, New York, New York; 5 Loyola University Chicago, Chicago, Illinois

## Abstract

**Objective:**

To determine the effectiveness of active, upper-room, germicidal ultraviolet (GUV) devices in reducing bacterial contamination in patient rooms in air and on surfaces as a supplement to the central heating, ventilation, and air conditioning (HVAC) air handling unit (AHU) with MERV 14 filters and UV-C disinfection.

**Methods:**

This study was conducted in an academic medical center, burn intensive care unit (BICU), for 4 months in 2022. Room occupancy was monitored and recorded. In total, 402 preinstallation and postinstallation bacterial air and non–high-touch surface samples were obtained from 10 BICU patient rooms. Airborne particle counts were measured in the rooms, and bacterial air samples were obtained from the patient-room supply air vents and outdoor air, before and after the intervention. After preintervention samples were obtained, an active, upper-room, GUV air disinfection system was deployed in each of the patient rooms in the BICU.

**Results:**

The average levels of airborne bacteria of 395 CFU/m^3^ before GUV device installation and 37 CFU/m^3^ after installation indicated an 89% overall decrease (*P* < .0001). Levels of surface-borne bacteria were associated with a 69% decrease (*P* < .0001) after GUV device installation. Outdoor levels of airborne bacteria averaged 341 CFU/m^3^ in March before installation and 676 CFU/m^3^ in June after installation, but this increase was not significant (*P* = .517).

**Conclusions:**

Significant reductions in air and surface contamination occurred in all rooms and areas and were not associated with variations in outdoor air concentrations of bacteria. The significant decrease of surface bacteria is an unexpected benefit associated with in-room GUV air disinfection, which can potentially reduce overall bioburden.

The recent epidemic waves have exposed the vulnerability of our social infrastructure to disease dissemination, and additional engineering controls focused on airborne pathogens are needed. The COVID-19 pandemic, a resurgence of endemic respiratory viruses [eg, respiratory syncytial virus (RSV), influenza, and rhinoviruses], and the increase in healthcare-associated infections (HAI) have highlighted the need for air disinfection in indoor environments. HAIs and multidrug-resistant organisms (MDROs)^1^ are also persistent indoor air problems that may be treated with germicidal ultraviolet (GUV) disinfection technologies, including upper-level, in-room UV and surface treatment systems.^2–5^ GUV disinfection technology has a long history of successful applications in both air and surface disinfection in hospitals, schools, government facilities, and commercial office buildings.^6^ When properly designed, GUV air-treatment systems can provide benefits in reduced incidence of respiratory disease, reduced asthma symptoms, and energy savings without harm to occupants.^7–13,34^

The US Environmental Protection Agency issued the Clean Air in Buildings Challenge in 2022 to encourage and support improving indoor air quality.^14^ Recommendations have included enhancing ventilation, air filtration, and adding GUV air-cleaning devices. The Centers for Disease Control (CDC) states that GUV can be a supplemental treatment to inactivate SARS-CoV-2 when options for increasing room ventilation and air filtration are limited.^4^ In addition, American Society of Heating, Refrigerating and Air-Conditioning Engineers (ASHRAE) Standard 170-2021 provides guidelines for filtration efficiencies, air changes per hour, and fresh air requirements for healthcare environments.^15^

Air disinfection may reduce the incidence of respiratory infection in office workers, school-aged children and others, and it may benefit burn trauma patients, for whom infections are common.^7–12^ Burn-related infections hamper wound healing, lengthenroom hospital stays, increase healthcare costs, and mortality rates can reach 33%–80%.^13–15^ Burn patients are at increased risk for HAIs as a result of the breakdown of the skin barrier and the profound systemic immunocompromising effects of the burn.^16^ In addition, the lengthy admissions of these patients make time-consuming terminal cleaning a challenge during the patient’s admission.

A previous study demonstrated that the installation of multiple fixed-in-room upper GUV air cleaners significantly reduced both airborne and surface-borne bacterial contamination in high-occupancy areas of public restaurants, offices, and media-production trailers.^17^ These GUV systems did not affect the supply air to the rooms, the flow rate of which remained unchanged throughout testing. In the current study, fixed-in-room GUV air cleaners were installed in multiple patient rooms in a hospital burn unit to test whether similar results could be obtained in a healthcare environment. Recently, our study site has seen a significant increase in *Candida auris* cases. The GUV system was installed as one of the mitigation measures to address this outbreak.

## Methods

This study was carried out at the Loyola University Medical Center Burn Intensive Care Unit (BICU) in Maywood, Illinois, over 4 months in 2022. The BICU is a 10-bed unit providing care to neonate, pediatric, and adult burn patients. The air disinfection system (UV Angel Clean Air, UV Partners, Grand Haven, MI) consists of a UV germicidal lamp (254 nm, 27 W UV output) enclosed within a high-intensity sealed chamber through which fans draw air through a MERV 7 filter. The total power draw for the unit is 115W. A constant airflow of 0.0236 m^3^/sec (50 cfm) airflow is exposed to the UV germicidal lamp inside the irradiation chamber during each pass, with an exposure time of ∼0.7 seconds. These units are integrated into a 2-foot (50-cm) × 4-foot (122-cm) light fixture installed in the ceiling. The unit weighs ∼22 pounds (∼10 kg).

The GUV in-room devices used in this study are not connected to the central heating, ventilation, and air conditioning (HVAC) air handling unit (AHU) and operate independently of all AHU controls. The advantage of the localized placement of GUV air-cleaning units is that contaminants remain in the room or area where they are released and are disinfected before migrating to other building areas. The GUV units utilized do not expose room occupants to UV due to the sealed chamber. Being fixed above the ceiling, the GUV air cleaners present no obstacles and are effectively invisible to occupants. They do not take up valuable floor space, and they operate automatically, requiring only annual lamp and filter replacement.

Air and surface samples were collected in various rooms throughout the day to establish a preinstallation baseline of contamination. Outdoor bacterial counts were measured as a reference. In each room, ∼11 preinstallation samples were collected. In addition, 10 postinstallation samples were collected from each room after installation of the GUV devices. All samples were obtained during normal hours of occupation without regard to the daily cleaning schedule. The ICU room size ranged from 175 square feet (16 m^2^) to 220 square feet (20.5 m^2^). All rooms had 9-foot (2.75 m) ceilings. Table 1 shows the room airflows and mean occupancy. Occupancy in the rooms was monitored before and after installation. Each occupancy number included the single patient and any attending healthcare providers when the sample was collected. Outdoor air samples were collected each morning and each afternoon for a total of 8 outdoor air samples: 4 before installation and 4 after installation of the UV Angel air units.

**Table 1.** Room Volume, Airflow, and Occupancy

Room/Area	Mean Occupancy	Floor Area, ft^2^	Volume, ft^2^	Airflow, cfm	ACH
Room 31	2.8	175	1575	493	18.8
Room 32	2.4	208	1872	438	14.0
Room 33	3.5	208	1872	450	14.4
Room 34	2.7	208	1872	450	14.4
Room 35	3.3	206	1854	463	15.0
Room 41	2.8	205	1845	439	14.3
Room 42	2.3	208	1872	510	16.3
Room 43	2.3	206	1854	418	13.5
Room 44	2.9	208	1872	510	16.3
Room 45	3.1	220	1980	427	12.9
Overall Average	2.8	205	1847	460	15.0

Note. cfm, cubic feet per minute.

All mitigated patient rooms had 2 GUV units. On average, the ratio of air treatment units to cubic feet was 1:990 cubic feet. After installation of the GUV systems, matched air and surface samples were retaken in the same locations. The central HVAC (AHU) system was reported to have a UV-C coil cleaning system in the AHU and MERV 14 air filters for the supply air. This system was reported by hospital facilities to be compliant with the requirements of ASHRAE 170 for this type of hospital unit.^15^ The air supplied by the central AHU was sampled to determine the CFU contribution delivered to the rooms. The hospital reported no change in cleaning procedures or AHUs before or during the study.

### Air sampling

Bacterial air samples were collected once per hour per occupied sample location over a 2-day, 16-hour period randomly within the patient rooms. In total, 100 air samples were obtained before installation, and 101 air samples were collected after the GUV device installation. The supply air was collected in the room following the same protocol using capture ventilation hoods. All patient rooms were sampled (room numbers 31–35 and 41–45). On average, before installation, most rooms were sampled 11 times over 16 hours. Only 1 preinstallation sample was collected from patient room 33 due to the medical condition of the patient in the room; the staff advised the technicians not to access that room. In the postinstallation sampling, all patient rooms were sampled 10 times over 16 hours. Each room was also sampled for the bacterial CFU counts from the supplied air in each sampling round. Preinstallation sampling occurred in March, followed by the GUV installation according to hospital infection control risk assessment (ICRA) protocols. Postinstallation sampling occurred in June. Environmental Molecular Services Laboratory (Houston, TX), processed all the sample plates.

An SAS 180 Air Sampler (Bioscience International, Rockville, MD) was used to sample the air. The D50 particle cutoff size for the SAS sampler was 1.51 µm. The aspirating head was cleaned with isopropyl alcohol and was allowed to dry before use. Sample plates (90 mm diameter) containing TSA with blood agar were inserted into the sampler, operated by a timer for ∼5.5 minutes, drawing 1,000 L of air. Samplers were positioned 1–1.5 m above the floor to collect samples in the breathing zone. The SAS sampler was in current calibration, and air sampling data (CFU counts) were corrected according to the SAS sampler instruction manual.^18^ Air bacteria concentrations were measured in colony-forming units (CFU) per cubic meter. The sampled CFU per 1,000 L (CFU/m^3^) were corrected according to the following equation:
(1)

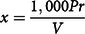




where x is the CFU/m^3^ of air; Pr is the probable count obtained by positive hole correction; and V is the volume of air sampled (1,000 L). The value of Pr was obtained from a correction table in the instruction manual.^18^ Because the maximum value of Pr =1,307 at a plate count of 219, any CFU values >219 were assumed to have a maximum value of Pr = 1,308.

Air sampling included collecting samples and metrics from the supply air vents, including viable bacteria counts, particle counts, relative humidity, temperature, and airflow in cubic feet per minute (cfm). Capture ventilation hoods were used to isolate the “clean” air supply to the room from the central AHU to collect bacteria levels. The preinstallation CFUs in the supply air were measured when no ceiling GUV devices were operating in the room, and levels were measured again when the ceiling GUV devices were operating in the patient rooms.

### Surface sampling

Surface samples were obtained using replicate organism detection and counting (RODAC) plates before and after installation in the same rooms and areas where air samples were collected. Some 99 preinstallation surface samples and 102 postinstallation surface samples were obtained from the 10 rooms. Surface samples were obtained from non–high-touch surfaces. The RODAC plates included tryptic soy agar supplemented with lecithin and tween to neutralize residues from chemical disinfectants. Firm pressure was applied to each plate for 30 seconds. Surface bacteria concentrations were measured in CFU per 25-cm^2^ plate, and values were summarized in units of CFU per plate. All surfaces sampled were identified as horizontal non–high-touch surfaces, such as the tops of shelves, tabletops, the top of televisions, headwall lights, rear area of sinks, etc. Postinstallation surface samples were matched at locations immediately adjacent to the locations where the preinstallation samples were collected.

### Particle counting

A model PCO-1 air sampler was used for particle counting.^19^ Particle counts were measured at 2.5 µm and 0.3 µm in all rooms and the outside air. In total, 197 particle-count samples were collected in the rooms before GUV device installation, and 4 control samples of the outside air were collected. In total, 212 particle-count samples were collected in the rooms after installation, and 4 control samples of the outside air were also collected.

### Statistical methods

Data were analyzed using statistical software NCSS version 11 software.^20^ Means, and standard deviations of data were analyzed using analysis of variance (ANOVA). Statistical significance was set at α = .05 to determine the *P* values for air samples, surface samples, and particle counts. Due to the nonnormality of the sample data and high variance, an Aspin-Welch unequal variance 2-sample *t* test was used for correlations.

## Results

**Figure 1.**
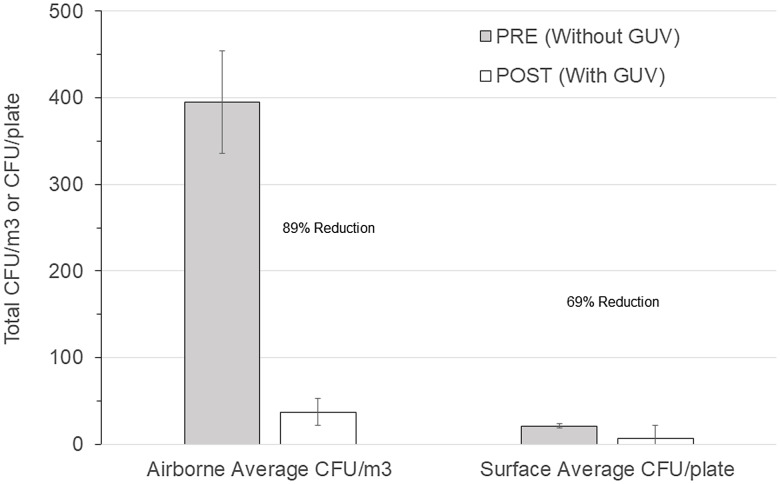
Comparison of before versus after overall room average colony-forming units (CFU) for germicidal ultraviolet light (GUV) in air (CFU/m^3^) and on surfaces (CFU/plate).

Overall airborne bacteria concentrations had a mean preinstallation value of 395 CFU/m^3^ (n = 100; standard error of the mean [SEM] = 12 CFU/m^3^) and a postinstallation value of 37 CFU/m^3^ (n = 100; SEM = 12 CFU/m^3^; *P* < .0001), representing a decrease of 89%. Room surface bacteria concentrations had a mean preinstallation value of 21 CFU/m^3^ (n = 100; SEM = 1 CFU/m^3^) and a mean postinstallation value of 7 CFU/m^3^ (n = 101; SEM = .6 CFU/m^3^; *P* < .0001), representing a decrease of 69%. Figure 1 illustrates these results along with the SEM. The outdoor air concentrations increased from an average of 341 CFU/m^3^ (n = 4; SEM = 323 CFU/m^3^) before installation to 676 CFU/m^3^ (n = 4; SEM = 365 CFU/m^3^) after installation, but this increase was not significant (*P* = .517). Table 2 summarizes the concentration reductions for the individual rooms and associated *P* values. The total airborne viable bacterial CFU before and after the installation of GUV air-cleaning devices (395 vs 37 CFU/m^3^, respectively) in the BICU represented an average reduction of 89% and a range of 76%–94% in the rooms.

**Table 2.** Average Mitigated CFUs for Airborne and Surface Samples With Aspin-Welch Unequal Variance *T*-Test *P* Values Before and After Installation of a UV-C Air Cleaning Unit

	Airborne Bacteria				Surface Bacteria			
	CFU/m^3^				CFU/plate			
Room/Area	Before	After	% Reduction	*P* Value	Before	After	% Reduction	*P* Value
31	382	38.2	90	.085	38	19	50	.073
32	381	27.1	93	.077	14	4	70	.010
33	135	32.5	76	.000	25	2	93	.000
34	344	74.3	78	.242	33	17	49	.437
35	489	28.1	94	.041	27	4	87	.020
41	492	29.8	94	.040	9	3	71	.208
42	606	56.4	91	.022	18	7	64	.047
43	380	28.1	93	.079	15	3	83	.029
44	372	21.4	94	.082	13	3	79	.003
45	376	36.4	90	.090	29	12	58	.036
Average	395	37	89	<.0001	21.1	6.5	69	<.0001

Note. CFU, colony-forming units.

**Figure 2.**
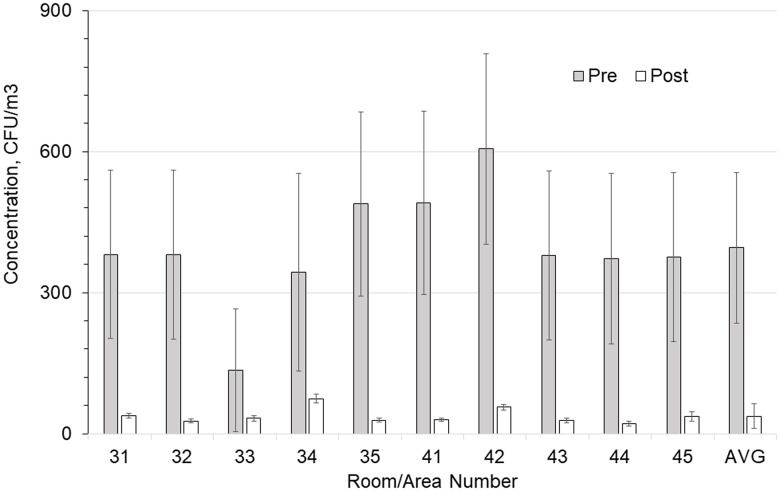
Comparison of total airborne colony-forming units (CFU) in each room with and without germicidal ultraviolet light (GUV) device. Error bars show the standard error of the mean (SEM) values.

**Figure 3.**
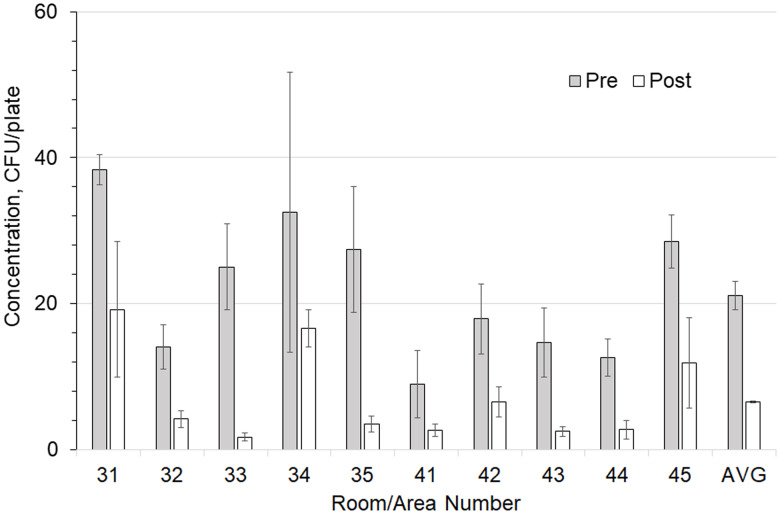
Comparison of total surface-borne colony-forming units (CFU) in each room with and without a germicidal ultraviolet light (GUV) device. Error bars show the SEM values.

Figures 2 and 3 show the preinstallation versus postinstallation airborne and surface concentrations for the individual rooms. No significant difference was seen in the bacteria counts of the air supplied to the room (Table 3). Room supply and exhaust airflows were monitored before and after the test and did not vary significantly. Supply airflows ranged from 10.6 to 14.4 m^3^/min and from 12.9 to 18.8 air changes per hour (ACH). Temperature and relative humidity were monitored throughout the test and remained at 59–82°F (15–28°C) and 19%–69%, respectively.

**Table 3.** Average Supply Hood CFUs for Airborne Samples With Aspin-Welch Unequal Variance *T* Test *P* Values Before and After Installation of a UV-C Air Cleaning Unit

	CFU/m^3^			
Supply Air	Before	After	% Change	*P* Value
Room 31	1.4	4.3	215	.324
Room 32	4.1	3.0	−27	.766
Room 33	1.0	0.6	−40	.000
Room 34	1.3	1.6	28	.722
Room 35	4.4	1.7	−61	.262
Room 41	1.7	2.4	39	.705
Room 42	3.5	1.6	−54	.242
Room 43	2.1	3.2	53	.665
Room 44	8.2	8.8	8	.925
Room 45	3.3	6.8	108	.530
Average	3.1	3.4	10.4	.964

Note. CFU, colony-forming units.

An average of 2.73 occupants per room per day before installation and 1.64 occupants per room per day after installation was not a statistically significant difference (*P* = .571). The CFU counts in the air supplied to the rooms, 3.1 CFU/m^3^ before installation and 3.4 CFU/m^3^ after installation, were very low compared to the CFU counts in the air in the room, 395 CFU/m^3^ before installation and 37 CFU/m^3^ after installation. These approximate proportions of supplied air remained consistent before and after GUV installation.

Particle counts showed large reductions between the pre- and postinstallation measurements (Table 4). For the smaller 0.3-µm particle sizes, the outdoor air percentage difference between preinstallation and postinstallation testing was 69% but was not statistically significant (*P* = .561). In contrast, the difference between the rooms was 56% with the GUV device (*P* = .048). For the larger 2.5-µm particles, the outdoor air change was 57% (*P* = .03). Particle counts with the GUV device in the rooms were 69% lower (*P* < .0001) overall. Surface sampling data before and after installation indicated an average 69% reduction in total settled surface bacteria CFU: 21 versus 7 (*P* < .0001).

**Table 4.** Particle Counts in Rooms and Supply Hoods Before and After Installation of a UV-C Air Cleaning Unit

Room/Area	0.3 µm				2.5 µm			
	Before	After	% Reduction	*P* Value	Before	After	% Reduction	*P* Value
Room 31	11,159	1,350	88	1.000	178.5	12.0	93	.149
Room 32	10,226	2,211	78	.321	206.5	35.0	83	.275
Room 33	2,800	1,689	40	1.000	13.0	14.2	−9	.268
Room 34	1,553	1,263	19	.446	13.0	10.3	21	.131
Room 35	1,967	1,356	31	.501	17.7	18.7	−5	.004
Room 41	1,903	1,198	37	.620	15.6	12.7	19	.032
Room 42	1,735	1,275	27	.640	18.8	11.7	38	.417
Room 43	2,363	1,478	37	.022	31.7	9.7	69	.170
Room 44	1,562	1,348	14	.915	11.4	10.2	10	.597
Room 45	1,915	2,224	-16	.915	15.1	23.0	−52	.072
Room average	3,526	1,550	56	.048	49	15	69	<.0001
Outside control	22,073	6,780	69	.561	141.3	61.3	57	.030
Supply air hoods	0.3 µm				2.5 µm			
Room 31	1,840	1,176	36	.304	7.0	2.2	69	.218
Room 32	1,403	1,086	23	.766	3.5	2.3	33	.181
Room 33	1,402	1,289	8	.870	2.0	4.2	−110	.874
Room 34	1,526	1,326	13	.133	5.7	3.4	41	.564
Room 35	1,564	1,214	22	.942	4.0	3.5	11	.217
Room 41	1,708	1,410	17	.055	8.0	4.0	50	.218
Room 42	1,892	1,128	40	.589	5.6	3.7	34	.170
Room 43	1,732	1,484	14	.100	5.4	3.6	33	.218
Room 44	1,578	1,138	28	.971	5.7	5.5	4	.038
Room 45	1,686	1,538	9	.314	6.0	6.6	−10	.192
Supply hood average	1,667	1,324	21	.005	6	4	31	<.0001

## Discussion

These results corroborate earlier studies on the same or similar GUV air disinfection devices in which airborne bacteria levels were significantly reduced.^17,21–24^ Previous studies have demonstrated that GUV can be effective at reducing airborne concentrations for a wide variety of pathogens, including *M. tuberculosis*, *Staphylococcus aureus*, *S. albus*, *E. coli*, *Streptococcus pneumoniae*, *Serratia marcescens, Pseudomonas aeruginosa*, *P. fluorescens*, *Proteus vulgaris*, *Moraxella catarrhalis*, influenza A, SARS-CoV-2 coronavirus, and various other pathogens that cause upper-respiratory infections.^8–10,12,13,23–33^

The result that air disinfection is significantly associated with the reduction of surface contamination is an important finding and corroborates previous studies. This important finding supports the concept that treating the air directly impacts surface contamination, where settled bacteria can be re-entrained by local air currents. Airborne bacteria settle out of the air at a rate that depends on their aerodynamic size.^6^ This result corroborates a previously published study that reported a 55% reduction in surface contamination with the same GUV air-cleaning system applied in commercial environments.^17^ GUV air disinfection may mitigate surface-borne disease transmission by reducing fomites, and some supportive evidence for this comes from testing of GUV devices in animal facilities wherein the level of transmission of nonrespiratory pathogens was reduced by UV air cleaning.^35^ This finding was also supported by a study conducted in a hospital ICU by Ethington et al,^21^ where a similar technology was associated with an overall 60% decrease in HAIs.

Although this air disinfection system does not exceed the surface reductions achieved by a dedicated terminal cleaning process, it removes a significant amount of settled surface-borne bacteria as a benefit of air disinfection. Continuous air treatment compliments the episodic patient room cleaning (routine and terminal). These are important considerations in a multilayered approach to infection prevention and control. Depending on the application, GUV technology may not be prohibitively expensive, but the question of cost may represent a study limitation; thus, cost-effectiveness should be considered in future research.

These results suggest that active, upper-room, GUV air disinfection can reduce levels of air and surface contamination and can diminish the total bioburden of the indoor environment. GUV can significantly reduce airborne concentrations in buildings in the same way that ventilation dilution reduces airborne concentrations—the addition of clean air to the building displaces contaminated air, and the indoor contaminant concentration decreases rapidly over time.

In high-density, high-contamination scenarios, or with high-risk patients, filtration and ventilation may not be enough. The reductions observed in this study of upper in-room GUV occurred in the setting of a pre-existing MERV 14 and UVC in the AHU and up to 18.8 ACH. Considering the AHU delivered, on average, 3 CFU/m^3^ of bacteria to the room, the high levels of CFUs in the patient room before the mitigation were due to people and patient care activities in the room. Installing the mitigation device at the point of care significantly reduced the viable bacteria levels in the patient room. The AHU alone did not reduce the viable bacteria load in this critical care area. This supplemental engineering control may improve the safety of highly susceptible patients following burn injury.

Our findings highlight the benefit of continuous versus episodic cleaning of the room, and these processes work together for a cleaner space. Patients in the BICU have lengthy admissions (the median BICU stay is 22 days). Continuous operation of the GUV system reduces the bioburden in this high-risk environment.

GUV active air-cleaning technology had a size-dependent effect on the particles in the room. Smaller particles of the 2.5-µm size range experienced significantly greater reductions in indoor air (69%) versus outdoor air (57%). The MERV 7 filter in the air disinfection unit may have also contributed to this decrease. More study is needed regarding minimizing particle concentrations by GUV air disinfection.

This study had several limitations. For example, the study duration was too short to measure any reductions in disease incidence, an area for much-needed future research. High-touch surfaces were not investigated. Our follow-up research will investigate the transmission of infections to patients and patient infection rates, and the cost-effectiveness of the application was not evaluated.

The data analyzed in this study suggest that in-room, active, supplementary, upper-room UV-C disinfection may decrease the concentration of bacteria in the air and on settled surfaces. The advantages of engineering control include (1) continuous operation, (2) not user dependent, and (3) safer environment of care, especially for those highly susceptible to cross infection and employees. Traditional air change rates may have limited effectiveness in critical care applications, such as BICU; thus, it may be time to consider supplemental environmental air cleaning technologies such as active GUV air disinfection.
